# Rapid Functionalization of Polytetrafluorethylene (PTFE) Surfaces with Nitrogen Functional Groups

**DOI:** 10.3390/polym13244301

**Published:** 2021-12-09

**Authors:** Alenka Vesel, Rok Zaplotnik, Gregor Primc, Miran Mozetič, Tadeja Katan, Rupert Kargl, Tamilselvan Mohan, Karin Stana Kleinschek

**Affiliations:** 1 Department of Surface Engineering, Jožef Stefan Institute, Jamova 39, 1000 Ljubljana, Slovenia; rok.zaplotnik@ijs.si (R.Z.); gregor.primc@ijs.si (G.P.); miran.mozetic@ijs.si (M.M.); 2 Institute for Chemistry and Technology of Biobased Systems, Graz University of Technology, Stremayrgasse 9, 8010 Graz, Austria; tadeja.katan@student.tugraz.at (T.K.); rupert.kargl@tugraz.at (R.K.); tamilselvan.mohan@tugraz.at (T.M.); karin.stanakleinschek@tugraz.at (K.S.K.)

**Keywords:** polytetrafluoroethylene, plasma treatment, surface functionalization, amination, etching, biocompatibility

## Abstract

The biocompatibility of body implants made from polytetrafluoroethylene (PTFE) is inadequate; therefore, the surface should be grafted with biocompatible molecules. Because PTFE is an inert polymer, the adhesion of the biocompatible film may not be appropriate. Therefore, the PFTE surface should be modified to enable better adhesion, preferably by functionalization with amino groups. A two-step process for functionalization of PTFE surface is described. The first step employs inductively coupled hydrogen plasma in the H-mode and the second ammonia plasma. The evolution of functional groups upon treatment with ammonia plasma in different modes is presented. The surface is saturated with nitrogen groups within a second if ammonia plasma is sustained in the H-mode at the pressure of 35 Pa and forward power of 200 W. The nitrogen-rich surface film persists for several seconds, while prolonged treatment causes etching. The etching is suppressed but not eliminated using pulsed ammonia plasma at 35 Pa and 200 W. Ammonia plasma in the E-mode at the same pressure, but forward power of 25 W, causes more gradual functionalization and etching was not observed even at prolonged treatments up to 100 s. Detailed investigation of the XPS spectra enabled revealing the surface kinetics for all three cases.

## 1. Introduction

Polymers are often used for the synthesis of vascular grafts because of their chemical and mechanical properties. A handful of different polymers were found useful for grafts, including polyethylene terephthalate, polyurethane, polytetrafluorethylene, silk and a variety of silicones [1,2,3,4,5]. All those materials exhibit different behaviors when in contact with human or animal blood. Generally, the non-treated polymers exhibit moderate hemocompatibility, which is reflected in activation of blood platelets, the surface transformation of blood proteins and non-controlled accumulation, the poor proliferation of vascular endothelial cells and rapid proliferation of soft muscle cells. All these effects may lead to serious complications such as thick neointimal formation, thrombosis and restenosis. The complications are a consequence of the inappropriate surface finish of the vascular grafts made from pristine polymers; therefore, the surface properties should be modified to suppress these unwanted effects [6,7,8]. Various techniques for modification of the surface properties have been reported in the scientific literature [9]; most of them involve the deposition of a thin film of bio-compatible materials.

The polymer of particular interest is polytetrafluorethylene (PTFE). Nojiri et al. [10] used a complex method for the deposition of various coatings on the PTFE grafts to obtain non-thrombogenic properties. The outer layer was polyethylene terephthalate, the middle polyurethane and the inner layer was an amphiphilic block copolymer composed of 2-hydroxyethyl methacrylate and styrene. The long-term in-vitro tests with model animals showed no thrombi formation, which was explained by irreversible adhesion of a very thin protein film on the inner side of the vascular graft. The rather complex structure with three layers was necessary for binding the active layer to the PTFE substrate.

Walluscheck et al. [11] coated vascular grafts made from expanded PTFE with bioactive heparin and reported a primary 1-year patency of approximately 90% and the 2-year patency was 70%. The authors reported that the influence of luminal heparin bonding was not only limited to thromboresistance, but also impacted the blood protein adsorption. No surface pre-treatment was reported.

Williams et al. [12] managed to deposit a thin film of laminin onto the inner surface of a PTFE graft with an inner diameter of only 1 mm. Covalent attachment of the laminin was reported despite no pre-treatment and histological evaluation confirmed the presence of endothelial cells on the midgraft lumenal surface of laminin-modified grafts. The endothelization was much faster than for untreated grafts.

Chong et al. [13] deposited polycarbonate urea urethane films onto PTFE substrates with incorporated polyhedral oligomeric silsesquioxane (POSS). The film was nanostructured by electron lithography and the final surface treatment was exposure to low-pressure oxygen plasma. The highly hydrophilic surface finish of the coating of rich morphology enabled improved adhesion and proliferation of endothelial vascular cells.

Wang et al. [14] used surfactants to improve the hemocompatibility of PTFE vascular grafts. Polyvinylamine was blended with pendent dextran and perfluoroundecanoyl branches. The deposition was performed by the dip-coating process. The authors reported spontaneous surface-induced adsorption and assembly of the coating on both smooth PTFE and expanded PTFE surfaces. The blood platelet adhesion was suppressed significantly as compared to non-treated materials.

Hunke et al. [15] treated PTFE powders in NH_3_ or H_2_ plasma. Significant defluorination was observed and attachment of polar oxygen moieties led to enhanced wettability. The thermal stability of the groups was studied up to 300 °C. Degradation of functional groups was observed; however, they did not completely degrade even at 300 °C. Surface ageing up to 12 months was investigated as well and the surface state was not completely reversible.

Gabriel et al. [16] used a wet chemical processing for the deposition of a biocompatible coating on the PTFE grafts. The adhesive peptide Arg-Glu-Asp-Val was deposited on previously activated surfaces using sodium naphthalenide. Excellent anti-thrombogenic properties were reported.

Ma et al. [17] reported a method for PTFE functionalization with hydroxyl groups using pre-treatment with benzoin. The PTFE membranes were then treated with chondroitin sulfate and an anticoagulant was grafted on the surface of the hydroxylated PTFE membrane using 3-aminopropyltriethoxysilane. As a result, enhanced proliferation of endothelial cells was reported.

Amine groups were successfully grafted onto PTFE surface by Υ-ray assisting technique as reported by Cocoletzi et al. [18]. In the first step, the acryloyl chloride was grafted using the direct gamma radiation method and in the second, a reaction between the grafted material and ethylenediamine was performed. A complex mechanism of the interaction between the acryloyl chloride and the PTFE substrate upon irradiation was proposed. The irradiation with energetic photons caused bond scission in the surface layer of PTFE and the dangling bonds were occupied with the acryloyl chloride molecules. The grafting efficiency was increased with the irradiation, but saturation was observed at the dose of about 15 kGy. The modification was not limited to the surface but also expanded to the sub-surface film, thus assuring for excellent adhesion of the film on the PTFE substrate.

The wet chemical techniques for activation of the PTFE surface reported by upper authors are hazardous for the environment, so many authors used plasma techniques as a pre-treatment prior to deposition of various coatings. Choi et al. [19] modified the surface properties of expanded PTFE by treatment with nitrogen ions of kinetic energy between 1 and 50 keV. The treatment caused both morphological and chemical changes of the polymer, but only minor changes in the biological response were reported.

Chandy et al. [20] reported various surface coatings on the PTFE grafts. As-received grafts were first treated with argon plasma and then coated with collagen and laminin. The coatings were found useful for immobilizing bioactive molecules like polyethylene glycol, heparin, or phosphatidylcholine via the carbodiimide functionalities. The plasma was sustained by a magnetized direct current (DC) glow discharge at the pressure of approximately 30 Pa. It assured an appropriate adhesion of the coating. As a result, the fibrinogen accumulation was suppressed to about 20% of the value typical for untreated grafts. The accumulation of blood platelets was reduced, too.

Cho et al. [21] used PTFE grafts treated with an atmospheric plasma sustained by dielectric barrier discharge. They invented a technique using hydrogen plasma pre-treatment, hydrocarbon deposition using a mixture of argon and acetylene and reactive plasma treatment in pure oxygen. The authors reported enhanced attachment of human cells on the surface of the PTFE grafts modified by this three-stage plasma processing.

Nakatani et al. [22] used a low-frequency RF discharge to sustain the plasma in methane at the pressure of 40 Pa. The vascular grafts made from PTFE were placed into the plasma reactor and homogeneous plasma was sustained inside the grafts. A layer of hydrogenated amorphous carbon was deposited by plasma-enhanced chemical vapor deposition (PECVD) and the neointimal formation was suppressed significantly as compared to untreated grafts.

Atmospheric pressure plasma was used for surface modification of PTFE by Nagatsu et al. [23]. The plasma jet was sustained in helium with the addition of ammonia. The substrates were biased negatively to enhance the functionalization with amino groups. Successive functionalization was attributed to the ion bombardment effect due to negative substrate bias. The bombardment created dangling bonds over the polymer surface and eventually promoted surface modification. They analyzed high-resolution C1s spectra using X-ray photoelectron spectroscopy (XPS) to confirm the breaking C-F bonds and creating C-C, C=O or C-N bonds. The high-resolution N1s spectrum revealed several nitrogen-containing functional groups. The surface concentration of amino groups introduced onto the PTFE surface was evaluated to be roughly 2 nmol/cm^2^. Such surface finish was found useful for the covalent bonding of several sulfur-containing biomolecules.

Bilek et al. [24] used atmospheric pressure plasma pre-treatment for covalent bonding of biomolecules on the PTFE surface. Plasma was sustained in ambient air by a dielectric barrier discharge. The static water contact angle decreased to about 75° after 2 s of plasma treatment and about 65° for prolonged treatment up to 15 s. The XPS analysis, however, showed a marginal surface functionalization with about 2 at.% of oxygen while the concentration of nitrogen was below the detection limit. Despite the rather marginal functionalization, such a surface finish was found useful for the good adhesion of bovine serum albumin (BSA) protein. The protein remained on the surface even after thorough washing.

The brief literature survey indicates a need for controlled functionalization of the polytetrafluoroethylene with nitrogen-containing functional groups. A standard technique for surface functionalization of polymers is a treatment with gaseous plasma. Depending on the plasma parameters, a variety of surface finishing was reported and the results of different authors are sometimes contradictory [25]. PTFE is particularly difficult to functionalize because the C-F bonds exhibit larger binding energy than C-N, so the substitution of fluorine in the surface film with nitrogen is an endothermic reaction [26].

The present paper reveals an alternative approach: instead of using single plasma treatment, we adopted a two-step plasma technique. In the first step, the PTFE surface film is depleted from fluorine and in the second step, ammonia plasma is used in order to graft nitrogen groups. The advantages and limitations of this technique are explained.

## 2. Materials and Methods

Samples of PTFE foils with a thickness of 0.5 mm were purchased from Goodfellow (Huntingdon, UK). The foils were cut to rectangular pieces and thoroughly cleaned with ethanol to remove any organic surface impurities. The samples were mounted into a plasma reactor immediately after the cleaning to suppress the adsorption of any molecules onto the surface.

The samples were treated in a low-pressure plasma reactor. The reactor was a 4 cm wide discharge tube made from borosilicate glass. The reactor was pumped with a two-stage rotary pump of a nominal pumping speed of 80 m^3^/h and ultimate pressure below 0.1 Pa. Inductively coupled radiofrequency (RF) plasma was sustained in the plasma reactor using an RF generator (Advanced Energy, Denver, CO, USA) operating at an industrial frequency of 13.56 MHz and adjustable output power up to 1500 W. A matching network enabled the optimization of the coupling between the RF generator and gaseous plasma. Plasma was sustained in hydrogen or ammonia gas (Messer, Ljubljana, Slovenia); both gases had a purity of 99.99%. The pressure was measured with an absolute gauge calibrated for the range between 1 and 1000 Pa. Plasma was characterized by optical emission spectroscopy (OES) using AvaSpec-3648 Fiber Optic Spectrometer (Avantes, Apeldoorn, Netherlands). The resolution of the spectrometer was 0.5 nm in the range of wavelengths between 200 to 1100 nm. The integration time was between 0.05 ms and 10 s.

Samples were probed by X-ray photoelectron spectroscopy (XPS) using apparatus TFA XPS (Physical Electronics, Munich, Germany). The samples were mounted in the XPS pre-chamber within a few minutes after the plasma treatment. The samples were excited with monochromatic Al Kα_1,2_ radiation at 1486.6 eV over an area with a diameter of 400 µm. Photoelectrons were detected with a hemispherical analyzer positioned at an angle of 45° with respect to the normal of the sample surface. Survey spectra were measured to determine the surface composition, i.e., the presence of any other elements except carbon. The survey spectra were measured at a pass energy of 187 eV with an energy step of 0.4 eV. The measured spectra were analyzed using MultiPak v8.1c software (Ulvac-Phi Inc., Kanagawa, Japan, 2006 from Physical Electronics), which was supplied with the spectrometer. Standard sensitivity factors were used for the calculation of the surface composition. High-resolution carbon peaks were fitted with six subcomponents positioned at the following binding energies: 285, 286.1, 287.9, 289.1, 292.2 and 293 eV.

## 3. Results and Discussion

**Figure 1:**
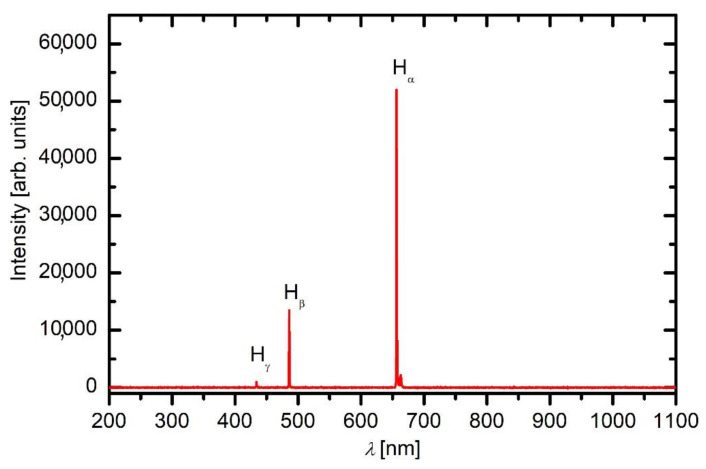
The optical spectrum during the treatment of PTFE with hydrogen plasma.

Individual samples were mounted into the plasma reactor. For each treatment, the reactor was evacuated by pumping with the rotary pump until the pressure was below the detection limit of the gauge (1 Pa). Hydrogen was introduced into the plasma reactor after achieving the ultimate pressure. Plasma was sustained in hydrogen for 1 s at the pressure of 35 Pa and forward RF power of 600 W. Such a short plasma treatment was used because the hydrogen plasma treatment causes depletion of fluorine from the surface film [27]. OES was used for basic plasma characterization during the polymer treatment with hydrogen plasma. A spectrum is shown in Figure 1. Only the Balmer series of atomic hydrogen is observed. The spectrum is free from molecular bands or continua, which indicates high dissociation of hydrogen molecules. The spectrum is also free from CH bands with the bandhead of 431 nm and CF_x_ bands or continua, which should have appeared in the near ultraviolet range of wavelengths [28]. The absence of such spectral features indicates marginal etching of the PTFE sample during such short plasma treatment.

**Figure 2:**
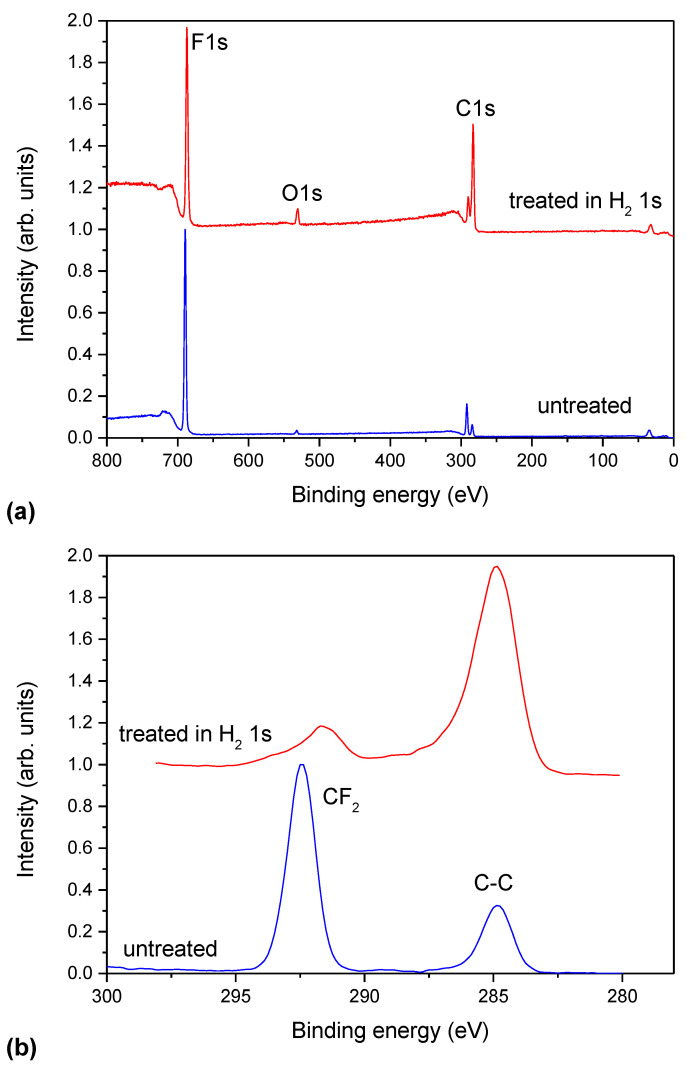
(**a**) The XPS survey and (**b**) high-resolution C1s spectra of an untreated sample and a sample treated with hydrogen plasma for 1 s.

The brief treatment of the PTFE sample with hydrogen plasma, however, causes a significant decrease in the fluorine concentration as probed by XPS. Figure 2a shows the survey spectra of the sample before and after the treatment with hydrogen plasma for 1 s, while Figure 2b shows the high-resolution C1s peaks for both samples. The survey spectrum for the untreated sample exhibits approximately 60 at.% of fluorine and 38 at.% of carbon. This composition deviates from the theoretical, i.e., 33 at.% C and 67 at.% F. There is a small peak of oxygen which is probably due to impurities adsorbed on the polymer surface after the cleaning and before the XPS characterization. The concentration of oxygen as revealed from the XPS survey spectrum of the untreated sample is 1.7 at.%. The high-resolution C1s peak (Figure 2b) explains the excessive carbon detected in the survey XPS spectrum of the untreated sample. Apart from the extensive peak at the binding energy of 292.5 eV, which corresponds to C-F_2_ group, there is also a weaker peak at 285 eV, which corresponds to organic impurities.

Hydrogen plasma treatment causes significant modification of the surface composition and structure. The XPS survey spectrum (Figure 2a) reveals a significant increase in carbon and a decrease in fluorine concentration. The carbon concentration is 66 at.%, whereas fluorine is 30 at.%. Approximately 4 at.% is the concentration of oxygen. The presence of oxygen is probably due to oxidation in ambient air after the treatment with hydrogen plasma. The high-resolution C1s peak for the hydrogen plasma-treated sample (Figure 2b) reveals a significant increase of the intensity of the peak at 285 eV, which corresponds to C-C and C-H bonds. The peak at 292.5 eV is much weaker than for the untreated sample and broadened to the lower binding energy of photoelectrons, indicating the change in the structure of the surface film. The changes as a result of hydrogen plasma treatment, as observed in Figure 2, are caused by the synergistic mechanisms between deep ultraviolet (UV) radiation and reactive hydrogen species (in particular H atoms). The radiation causes breakage of the surface bonds, leading to depletion of fluorine and the reactive hydrogen species occupy the dangling bonds.

The samples pretreated with hydrogen plasma were further treated with ammonia plasma without breaking the vacuum conditions. Once the hydrogen plasma treatment was accomplished, the reactor was pumped to the ultimate pressure and ammonia was introduced. Plasma was sustained in ammonia at the pressure of 35 Pa for various treatment times. Each treatment was accomplished with a fresh sample—a sample was installed in the plasma reactor, first treated with hydrogen plasma, followed by ammonia plasma treatment and then characterized by XPS. Treatment with ammonia plasma was performed at three different conditions: (1) in the continuous mode at the forward power of 200 W, (2) in the pulsed mode at the forward power of 200 W with the plasma-on and off time of 10 and 60 s, respectively and (3) in the continuous mode at the forward power of 25 W.

**Figure 3:**
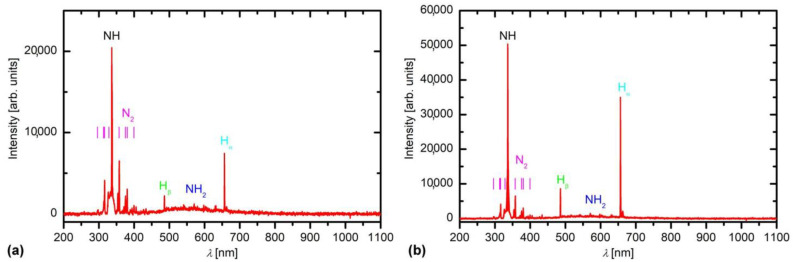
The optical spectrum during the treatment of a PTFE with ammonia plasma at the power of: (**a**) 200 W and (**b**) 25 W. The integration times were 0.05 and 10 s, respectively.

Optical spectra of ammonia plasma were acquired during the treatment. A typical spectrum at the forward power of 200 W is shown in Figure 3a. The integration time was 50 ms. The most intensive radiation arises from NH radicals, followed by the Balmer series of the atomic hydrogen transitions. The spectrum in Figure 3a, therefore, indicates significant dissociation of ammonia upon plasma conditions at the forward power of 200 W. There is also a weak NH_2_ continuum in the range of wavelengths between about 450 and 700 nm. The nitrogen band in the near UV range (roughly between 300 and 400 nm) indicates the presence of excited neutral nitrogen molecules. The molecules are probably formed at heterogeneous surface recombination of N atoms to parent molecules on the surface facing the plasma.

An optical spectrum of ammonia plasma at the forward power of 25 W is shown in Figure 3b. Because of the weak plasma radiation, the integration time was much longer at 10 s, so the intensities in Figure 3a,b are not comparable. Interestingly, the spectra look similar, except that the ratio between the intensity of spectral features is different. At the low discharge power (Figure 3b), the relative intensity of the NH_2_ band is much stronger than at the power of 200 W. Here, it is worth mentioning that the inductively coupled plasma appears in two distinguished modes: the E- and the H-mode. Low radiation is typical for the E-mode because of its small electron density. Plasma in the E-mode is further characterized by a rather high electron temperature because of the capacitive character of the coupling. The electrons are accelerated in the sheath between the powered RF coil and plasma so they gain significant energy. In contrast, the plasma in the H-mode is characterized by large electron density and lower electron temperature. In our case, plasma is in the H-mode at the discharge power of 200 W and in the E-mode at the power of 25 W. Detailed description of the discharge modes in ammonia plasma is provided in [29].

**Figure 4:**
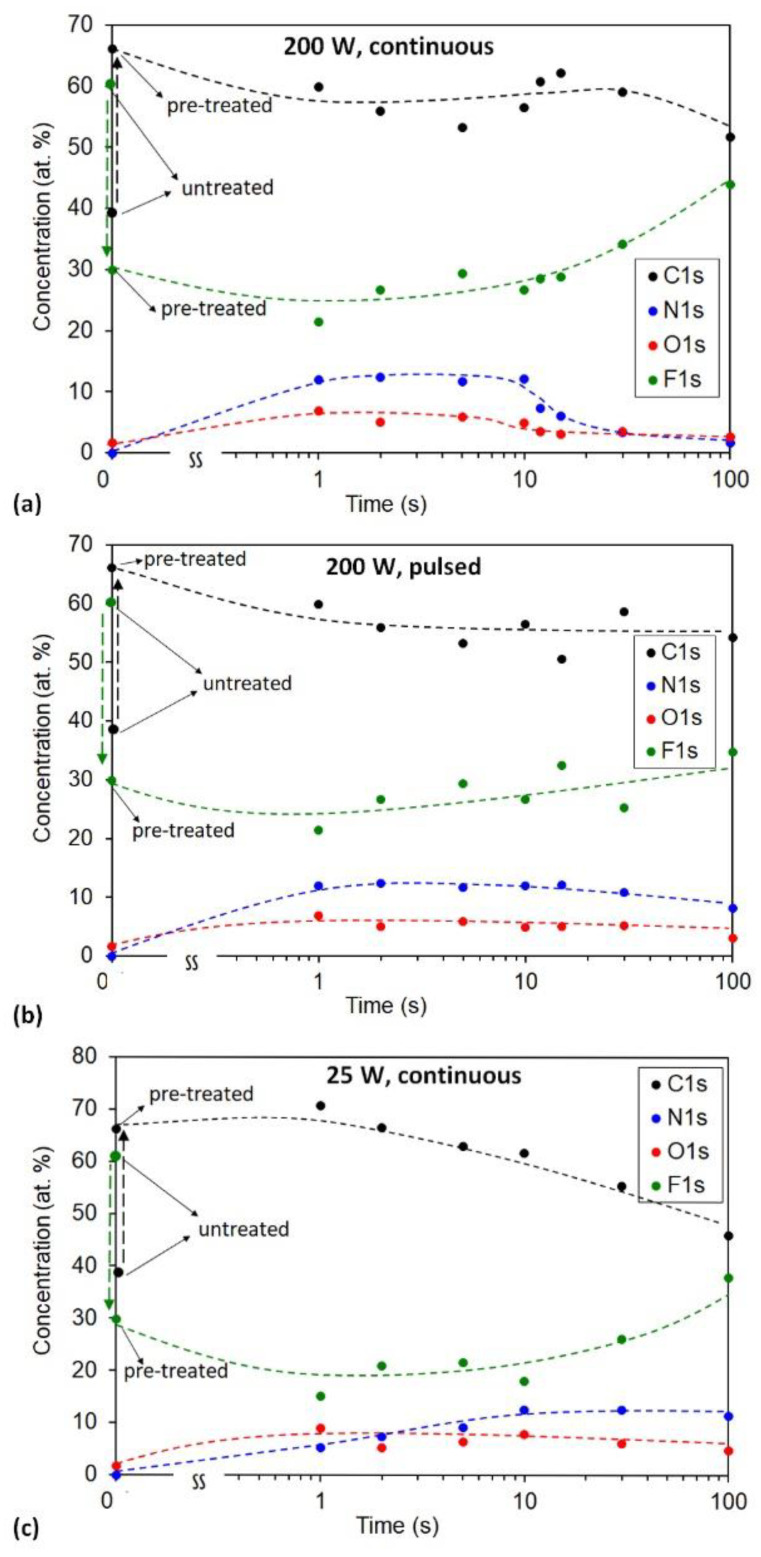
The XPS surface composition versus treatment time in ammonia plasma: (**a**) 200 W, continuous treatment (**b**) 200 W, pulsed treatment and (**c**) 25 W, continuous treatment. All samples were pretreated with hydrogen plasma at 35 Pa, 600 W for 1 s.

The significant dissociation of ammonia gas upon plasma conditions assures functionalization of the polymer surface with nitrogen-containing functional groups. XPS characterization was used to examine the effect of ammonia plasma treatment. The atomic surface composition versus treatment time is shown in Figure 4. All samples were pretreated with hydrogen plasma for 1 s as described above. Therefore, time 0 s corresponds to the sample pretreated with H_2_ plasma for 1 s. As a reference, the composition of untreated PTFE is also shown. The treatment with ammonia plasma in the continuous mode causes a rapid functionalization of the surface film with nitrogen groups (Figure 4a). A second of plasma treatment at 200 W is sufficient to obtain 12 at.% of nitrogen in the surface film probed by XPS. Increasing treatment time has little effect on the nitrogen concentration up to 10 s. Further treatment, however, causes a significant decrease in the N concentration: at 15 s, the concentration is 6 at.% and at the longest treatment time of 100 s, it is solely 2 at.%. Such behavior is explained by the etching of the surface film (previously modified with hydrogen plasma) with ammonia plasma. This is the reason for the upward deflection of the curve showing F concentration at longer treatment times. The etching of fluorine-depleted film increases with increasing treatment time because the sample temperature is elevated due to the surface neutralization of charged particles and weak bombardment of the sample with hydrogen ions. As explained above, ammonia plasma is in the H-mode at the discharge power of 200 W, so the density of electrons is rather large. For example, Fantz et al. [30] reported the density of charged particles of the order of 10^18^ m^−3^ in plasma sustained at conditions practically identical to ours.

If significant heating of the polymer sample is the reason for etching and, thus, decrease in the nitrogen concentration on the surface of PFTE after prolonged treatment time, the effect should be suppressed by using pulsed treatments. According to the results obtained with continuous treatment, the tolerable duration of the pulse should be 10 s. The next set of experiments was therefore performed with the plasma in pulses of 10 s and the off-time between subsequent pulses of 60 s. The evolution of the surface composition and nitrogen concentration in the surface film when pulsed treatment was performed is presented in Figure 4b. One can observe only a slight decrease in the N concentration with a long treatment time. A slight decrease of N concentration thus correlates with a slight increase of F concentration. The results indicate that such a pulsed treatment suppresses the etching but does not completely eliminate it because the N concentration keeps decreasing at prolonged treatment times.

Another set of experiments was performed at the forward power of 25 W when plasma was in the E-mode. The results are plotted in Figure 4c. The behavior is now different: the N-atom concentration slowly increases with treatment time until saturation is observed at 10 s. The rather slow increase in the N concentration in the initial 10 s of treatment with a weak ammonia plasma is explained by a shortage of reactive species in plasma at 25 W, as compared to powerful plasma at 200 W.

In Figure 4, we can also observe some oxygen in the samples, which may be due to the presence of water vapour in the vacuum system or because of exposure of the treated sample to the atmosphere prior to characterization.

**Figure 5:**
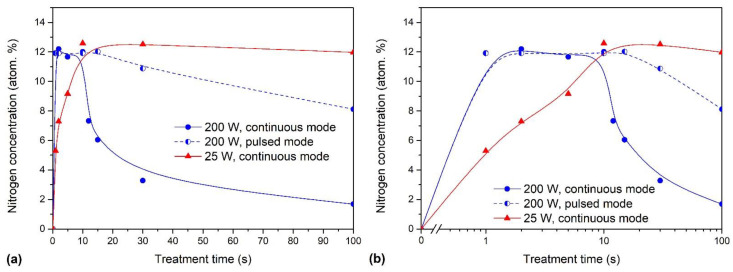
The concentration of nitrogen versus treatment time in ammonia plasma: (**a**) linear and (**b**) logarithmic scale. All samples were pretreated with hydrogen plasma at 35 Pa, 600 W for 1 s.

For the sake of clarity, we summarized the results for nitrogen concentration from Figure 4 in Figure 5. Figure 5a represents the evolution of the nitrogen concentration with the linear scale of the x-axis, while Figure 5b with the logarithmic scale. The results summarized in Figure 5 provide a recipe for the successful functionalization of hydrogen plasma pretreated PTFE samples with nitrogen functional groups. If a rapid functionalization is a goal, the powerful plasma of short duration (of the order of a second) should be applied. If longer treatment times are tolerable, the saturation of the surface film with nitrogen functional groups is also achievable. Interesting enough, the saturation concentration of the nitrogen in the surface film as probed by XPS is always approximately 12 at.%, irrespective of the discharge power.

**Figure 6:**
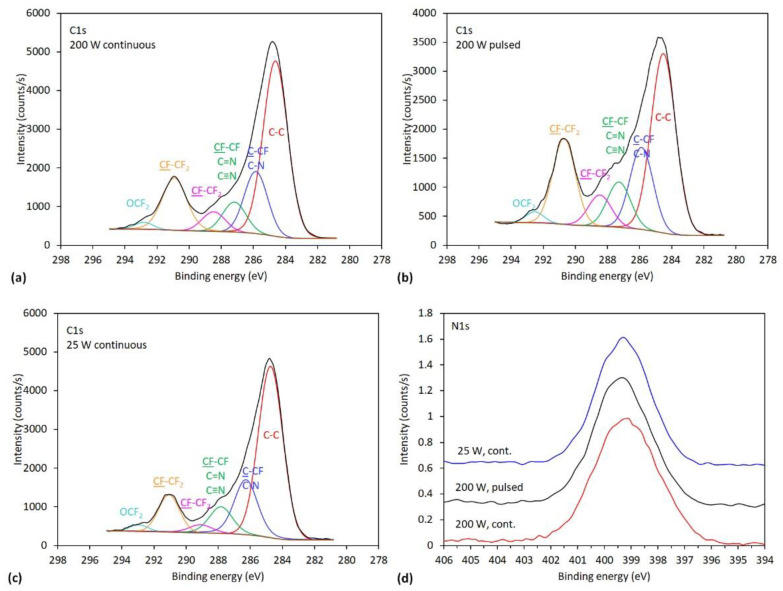
The XPS high-resolution spectra of C1s of the samples treated for 10 s with subcomponents at: (**a**) 200 W in continuous mode, (**b**) 200 W in pulsed mode, (**c**) 25 W in continuous mode and (**d**) comparison of corresponding N1s peaks.

Apart from the composition, the treatment with ammonia plasma also causes chemical structural changes in the surface film of PTFE samples. The chemical structure is revealed by examining the high-resolution XPS spectra. In Figure 6a–c are shown examples of C1s peaks and Figure 6d is shown a comparison of N1s peaks for the samples treated at 10 s, where the concentration of nitrogen was maximal, as revealed from Figure 5. Carbon peaks can be fitted with six subpeaks that were attributed to C-C/C-H, C-CF and C-N, CF/C-N/C=N/C≡N, CF-CF_2_, CF_2_-CF and OCF_2_ groups, respectively [31,32,33]. It should be mentioned that in addition to these groups, there may also be some oxygen groups present, which overlap with the aforementioned groups, but oxygen groups are not the subject of interest in this investigation. It should also be mentioned that the presence of fluorine atoms causes chemical shifts also at their neighbouring atoms; therefore, the exact interpretation of fluorine compounds is not trivial. Furthermore, for nitrogen functional groups, only small chemical shifts are observed; therefore, it is not possible to draw reliable conclusions about the chemical configurations of nitrogen (amines, imines or even nitriles) [32]. Due to the completeness of the paper, we show in Figure 6d also a comparison of N1s peaks. More information regarding nitrogen can be found in Supplementary Material in Figure S1.

**Figure 7:**
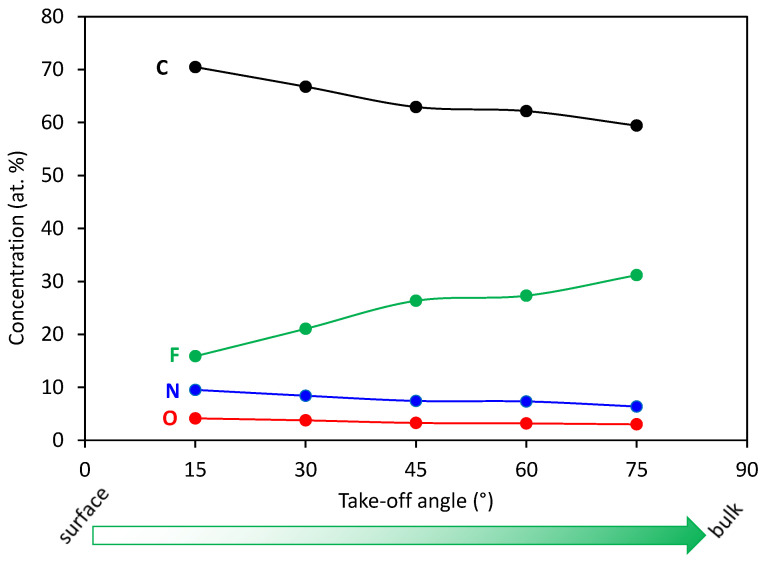
Variation of the surface composition of the sample treated for 10 s at 200 W versus the take-off angle (i.e., different detection depth).

**Figure 8:**
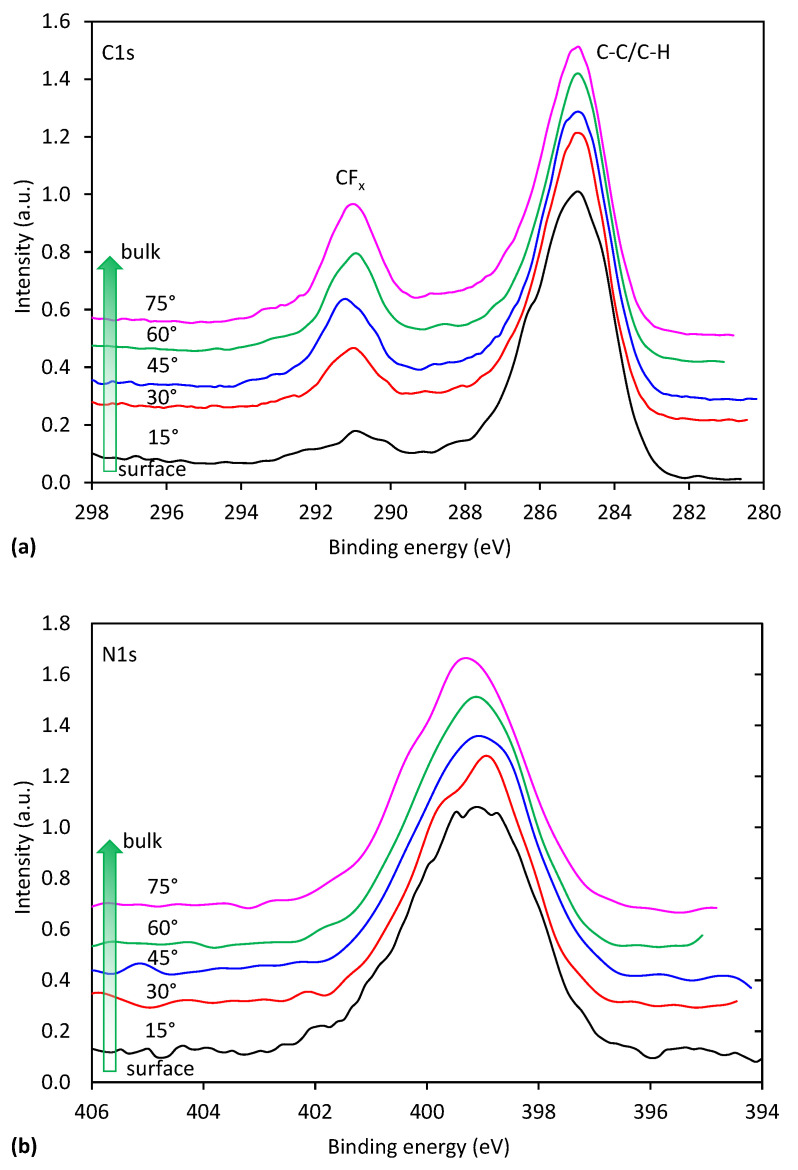
Evolution of high-resolution peaks of: (**a**) carbon and (**b**) nitrogen with detection depth.

In order to get more information regarding the evolution of the functional groups on plasma-treated surfaces, we also performed angle-resolved XPS measurements (AR-XPS). In Figure 7 is shown the composition of the selected sample with the maximum nitrogen concentration versus the take-off angle, whereas in Figure 8a,b is shown a comparison of carbon and nitrogen peaks recorded at different take-off angles. In Figure 7, we can observe that fluorine concentration is gradually increasing with increasing detection depth, being the lowest at the surface. Such a result is expected for plasma-treated samples because it is known that plasma modification is limited to the surface region only. Variations of fluorine concentration with detection depth are reflected also in high-resolution spectra shown in Figure 8. Consistently with Figure 7, we can observe in Figure 8a for the spectrum recorded more at the surface a pronounced C-C/C-H peak accompanied with only a minor peak corresponding to CF_x_ species. With increasing detection depth, the CF_x_ peak is increasing and becoming more and more pronounced. However, the CF_x_ peak for the spectrum recorded at the largest detection depth is still smaller than for the case of the unmodified PTFE (Figure 2b), indicating that the thickness of the modified layer is larger than the detection depth of XPS method because of larger penetration depth of VUV photons.

**Figure 9:**
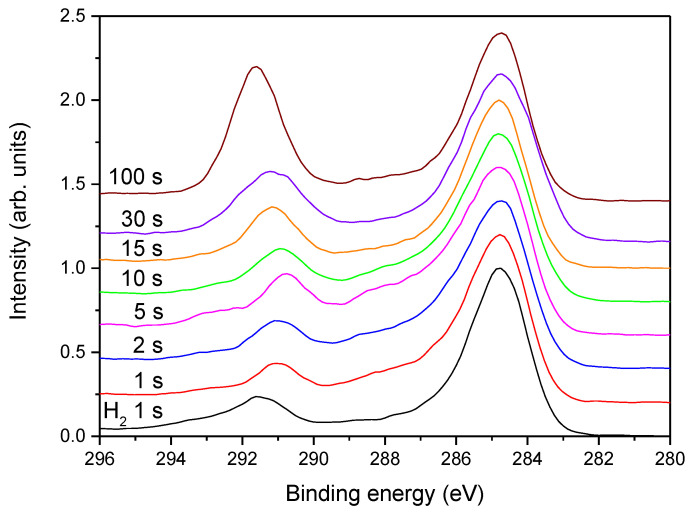
The high-resolution C1s spectra for samples treated at different times in ammonia plasma in the continuous mode at the power of 200 W.

**Figure 10:**
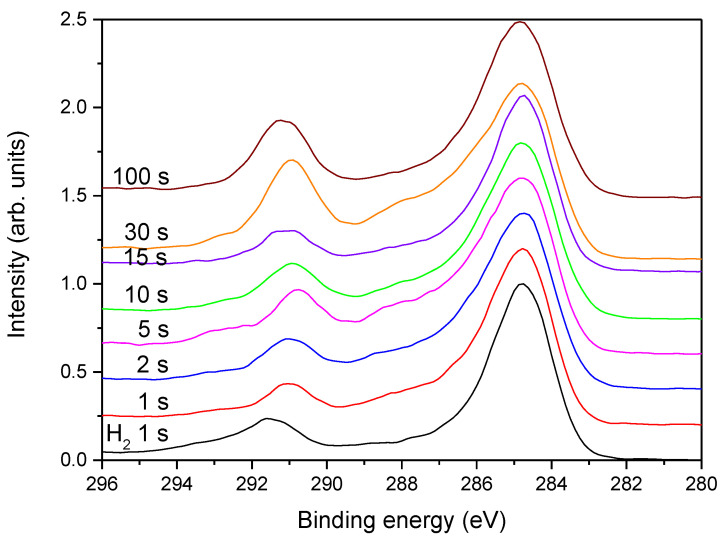
The high-resolution C1s spectra for samples treated at different times in ammonia plasma in the pulsed mode at the power of 200 W.

**Figure 11:**
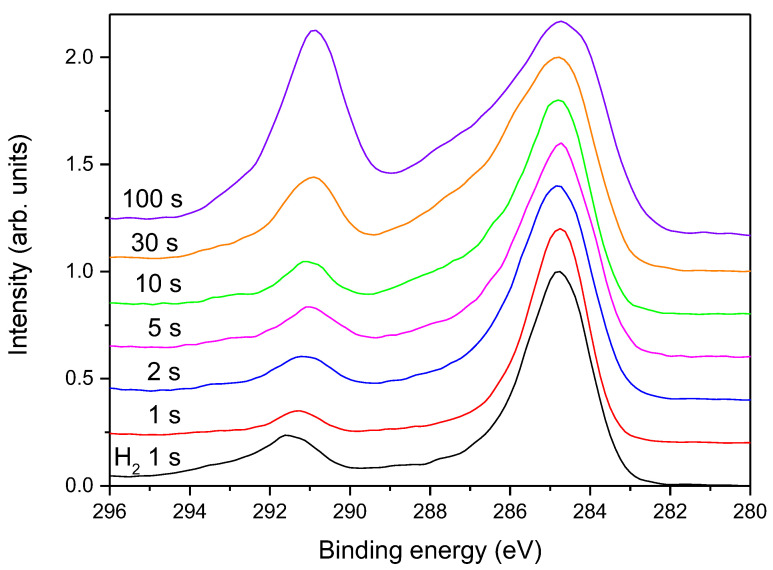
The high-resolution C1s spectra for samples treated at different times in ammonia plasma in the continuous mode at the power of 25 W.

In Figure 9 is shown the evolution of high-resolution C1s spectra for samples treated at different times in the continuous mode at the power of 200 W, whereas in Figure 10, spectra are shown for the pulsed mode at 200 W and in Figure 11 for the continuous mode at 25 W. The high-resolution C1s peak for a sample treated only with hydrogen plasma is added to all these figures for comparison.

Figure 9 reveals the XPS C1s peaks for samples treated in ammonia plasma in the continuous mode at the forward power of 200 W. The shortest treatment time of 1 s causes a further shift of the original peak at 292.5 eV, which corresponds to CF_2_ group, as compared to the sample treated with hydrogen plasma only. The shift is not dramatic but clearly distinguished. Therefore, even a second of treatment with a powerful ammonia plasma causes further degradation of the original polymer structure. As indicated by a comparison of the OES spectra of hydrogen (Figure 1) and ammonia (Figure 3a) plasmas, the ammonia plasma is also a source of radiation arising from H atoms. The radiation causes further degradation of the original polymer. Since the peak is shifted to lower photoelectron binding energy (peaked at approximately 291.5 eV), it means that the concentration of CF_2_ groups in the surface film as probed by XPS after the ammonia plasma treatment for 1 s dramatically decreased at the expense of the increase of C-F bonds. Simultaneously with the shifting of this C-F_x_ peak, the asymmetry of the peak at 285 eV becomes more obvious. The asymmetry is due to the chemical bonding of nitrogen to carbon in the surface film as probed by XPS. Further treatment with ammonia plasma causes an increase in the high energy peak at 291.5 eV and also a slight shifting towards high photoelectron energy. The effect increases with increasing treatment time and is most pronounced at the longest treatment time of 100 s. The original PTFE structure is not completely re-established, but the C1s XPS spectrum for the longest treatment time in Figure 9 is not much different from the spectrum of an untreated sample, which is shown in Figure 2b. The re-establishment of the original PTFE structure is explained by the etching of the film which has been previously modified by the treatment with hydrogen plasma. The fluorine-depleted film as obtained by hydrogen plasma treatment is not enriched with fluorine but rather etched as a consequence of the prolonged ammonia plasma treatment. Simultaneously with the increasing intensity of the high-energy peak, the intensity and asymmetry of the low-energy peak are weakened, indicating the loss of chemically bonded nitrogen in the surface film. This observation is based on the examination of the results in Figure 9 and is in agreement with the curve for continuous treatment at 200 W in Figure 5.

Figure 10 represents the evolution of the C1s peaks upon treatment with ammonia plasma at 200 W and pulsed mode. The results are similar to those observed in Figure 9 for short treatment times but deviate at longer treatment times. The intensity of the high-energy peak in Figure 10 at the longest treatment time of 100 s is significantly different from the corresponding peak observed after the treatment in the continuous mode (Figure 9). The discrepancy is sound with the results presented in Figure 5: the pulsed-mode plasma does not cause as extensive etching as the plasma in the continuous mode, so nitrogen still persists in the surface film even after 100 s treatment with ammonia plasma in the pulsed mode.

**Figure 12:**
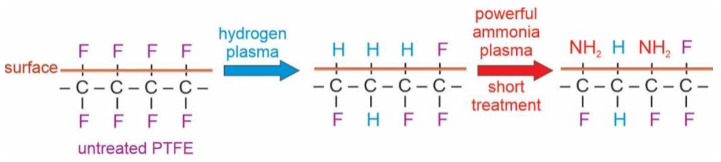
Simplified illustration of the effect of ammonia plasma sustained at the large power and short treatment times.

**Figure 13:**
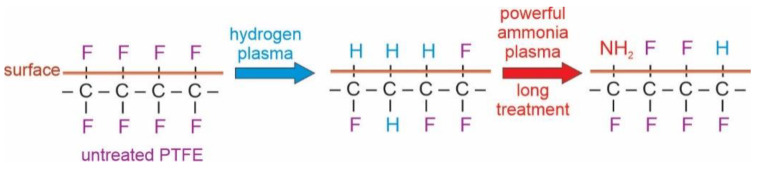
Simplified illustration of the effect of ammonia plasma sustained at the large power and long treatment times.

**Figure 14:**
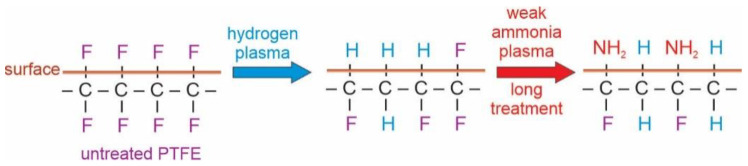
Simplified illustration of the effect of ammonia plasma sustained at the small power and long treatment times.

Interesting results are observed in Figure 11, which represents the evolution of the C1s peak upon treatment of the hydrogen plasma pretreated sample with ammonia plasma at the forward power of 25 W. According to the upper discussion and the results in Figure 5, one would expect persistence of the rather low intensity of the high energy peak. However, the high-energy peak in Figure 11 keeps increasing with increasing treatment time. The paradox is explained by a detailed investigation of the high-energy peak. As explained above, the high-energy peak in the case of treatments with powerful ammonia plasma keeps shifting to higher energy with increasing treatment time. In contrast, the high energy peak in Figure 11 remains at the photoelectron energy of 291.5 eV. This observation indicates that the fluorine-depleted surface film remains on the surface of hydrogen plasma pretreated samples even after the longest treatment time in the weak ammonia plasma. Furthermore, the asymmetry of the peak at 285 eV is preserved in Figure 11 even for the longest treatment time of 100 s. The effect of the weak ammonia plasma is, therefore, significantly different from the powerful plasma: instead of etching, the fluorine-depleted surface film persists and it consists of carbon atoms bonded to either fluorine or nitrogen. The surface carbon atoms are thus, functionalized with both F and N atoms when using a weak ammonia plasma. The effect of ammonia plasma treatment at large and small forward powers is illustrated in a simplified manner in Figure 12, Figure 13 and Figure 14.

Figure 12, Figure 13 and Figure 14 are simplified because the plasma treatment does not cause modification of the surface properties alone, but rather a thin film of the thickness of several nm, same as the escape depth of photoelectrons. Still, the effects can be explained as follows: Powerful hydrogen plasma is an extensive source of both reactive hydrogen species [34] and deep UV radiation [30]. The deep UV radiation causes bond scission and this depletion of fluorine in the surface film. After the hydrogen plasma treatment, the surface film contains carbon which is bonded predominantly to hydrogen. Such a surface film resembles polyolefins which may be functionalized with appropriate functional groups rather easily because the C-H surface groups are substituted with NH_2_ groups upon the treatment with ammonia plasma [35,36]. The functionalization kinetics depend on the density of NH and/or NH_2_ radicals in ammonia plasma. As long as ammonia plasma is in the E-mode (low forward power of 25 W in our case), the functionalization is rather slow; therefore, it takes about 10 s to saturate the surface with nitrogen-containing groups. When ammonia plasma is in the H-mode, however, the NH_3_ molecules are dissociated well, so the saturation of the N-containing functional groups appears in a second of plasma treatment.

As already mentioned, the exact nature of the nitrogen chemical bonds in the surface film as probed by XPS cannot be determined from high-resolution C1s peaks because of overlapping with fluorine groups. In addition, examination of the N1s peak can not give a clear answer because the chemical shift between different nitrogen functional groups is too small to enable reliable conclusions. Some authors use derivatization to distinguish between primary and secondary amines, nitriles and imines [37,38,39]. The technique is difficult to apply and is beyond the scope of this paper. Furthermore, derivatization is performed by using chemical agents containing a marker element that should not be present in the investigated material. Unfortunately, as a marker, often agents containing fluorine atoms are used, which are also present in our samples. For detection of nitrogen functionalities, the following markers are used trifluoroacetic anhydride (TFAA) and 4-trifluoromethyl benzaldehyde (TFBA) [40]. However, it was reported that these substances could react with amines as well as with imines; therefore, they are not selective enough [41,42]. Recently, Manakhov et al. [43] reported derivatization using 5-iodo 2-furaldehyde to reveal the concentration of amino groups on the surface of complex polymers such as 3-aminopropyl triethoxy silane and cyclo-propylamine deposited by plasma polymerization [44]. The derivatization enabled reliable results, but the procedure is not trivial.

## 4. Conclusions

The functionalization of polytetrafluorethylene with nitrogen-containing functional groups was accomplished using a two-step plasma treatment. In the first step, the samples were treated with hydrogen plasma to deplete the surface film from fluorine. The samples were exposed to ammonia plasma in the second step to functionalize the surface film with nitrogen-containing groups. The functionalization kinetics were revealed from the survey and high-resolution XPS peaks. The results showed that the maximal concentration of nitrogen in the surface film as probed by XPS was 12 at.%, irrespective of the plasma parameters. The plasma treatment time, which was useful for surface saturation with the N-containing functional groups, however, depended on the discharge mode. A range of treatment times between approximately 1 and 10 s was found appropriate for functionalization when ammonia plasma was in the H-mode and the treatment was accomplished in the continuous mode. The useful range of treatment times was expanded in the case when plasma in the H-mode was applied in the pulsed mode. When weak ammonia plasma in the E-mode was applied, the functionalization took a long time and the saturation in the nitrogen concentration in the surface film as probed by XPS appeared after about 10 s. The high N concentration was preserved even after 100 s of plasma treatment in the E-mode. The results are useful for a broad community working on modifying polytetrafluorethylene materials for covalent coating with various biocompatible materials, such as PFTE vascular grafts or prostheses.

## Data Availability

The data presented in this study are available on request from the corresponding author.

## Supplementary Materials

The following are available online at https://www.mdpi.com/article/10.3390/polym13244301/s1, Figure S1: High-resolution N1s spectra of PTFE sample treated in NH_3_ plasma for 10 s at: (**a**) 200 W in continuous mode, (**b**) 200 W in pulsed mode and (**c**) 25 W in continuous mode. The spectra were fitted with two components at binding energies of 398.9 eV and 399.8 eV.
