# Supplementary material for: Rapid Functionalization of Polytetrafluorethylene (PTFE) Surfaces with Nitrogen Functional Groups
Source: Polymers (Basel). 2021 Dec 9;13(24):4301. doi: 10.3390/polym13244301 (PMC8708819; doi:10.3390/polym13244301)
Supplement: Supplementary file 1 [file polymers-13-04301-s001.zip › polymers-1478781-supplementary.pdf]

polymers

MDPI

Supplemental information

Rapid Functionalization of Polytetrafluorethylene (PTFE) Surfaces with Nitrogen Functional Groups

Alenka Vesel [1,*], Rok Zaplotnik [1], Gregor Primc [1], Miran Mozetič [1], Tadeja Katan [2], Rupert Kargl [2], Tamilselvan Mohan [2] and Karin Stana Kleinschek [2]

In Figure S1 is shown an example of nitrogen peak fitted with two components positioned at 398.9 eV and 399.8 eV, that we attributed to C-N and to C=N/C≡N as reported by Willson also [32] and as also expected for plasma-treated samples. Here we should also mention that the presence of N-C=O can not be excluded; however, the oxygen concentration in our samples is very small, thus the contribution of N-C=O should be minor. Furthermore, we can also expect that oxygen could also be bound to carbon-oxygen functional groups only. The fitting of N1s peak is not straightforward, because the nitrogen shifts are too small to separate the peaks and because different values are reported for binding energies in the literature where the values scatter a lot.

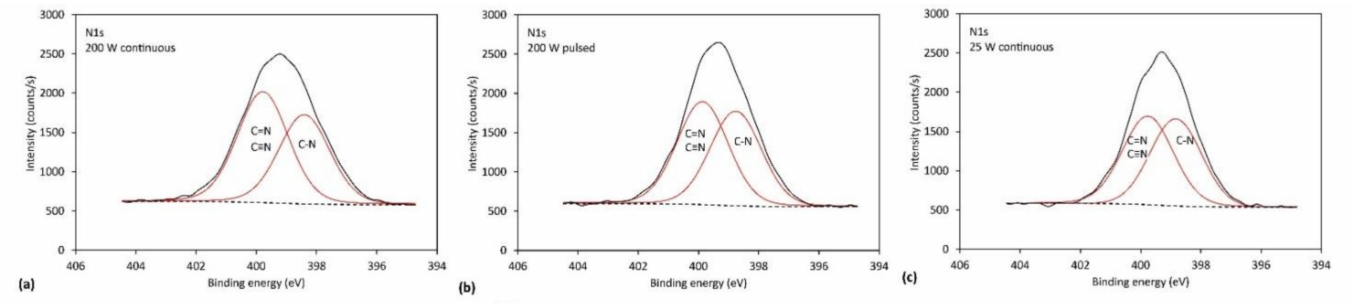

Figure S1. High-resolution N1s spectra of PTFE sample treated in NH_3 plasma for 10 s at: (**a**) 200 W in continuious mode, (**b**) 200 W in pulsed mode, and (**c**) 25 W in continuious mode. The spectra were fitted with two components at binding energies of 398.9 eV and 399.8 eV.
